# Editorial: Novel insights into syndromic micrognathia: from pathogenesis to clinical management 2023

**DOI:** 10.3389/fped.2024.1387539

**Published:** 2024-05-17

**Authors:** Weimin Shen

**Affiliations:** Department of Burns and Plastic Surgery, Children’s Hospital of Nanjing Medical University, Nanjing, China

**Keywords:** distraction osteogenesis, children, upper airway obstruction, nutrition status, complications FNMA, MNMA, MMFA, FMA

**Editorial on the Research Topic** Novel insights into syndromic micrognathia: from pathogenesis to clinical management 2023

Micrognathia, one of the most common craniofacial malformations, is characterized by unilateral or bilateral hypoplasia of the mandible. The study and treatment of syndromic micrognathia in infants is still challenging and requires both highly competent surgeons and airway teams. This research topic “Novel Insights into Syndromic Micrognathia: from Pathogenesis to Clinical Management 2023” received interest from authors from different geographical regions. Seven articles were included in this research topic.

The pathogenesis of syndromic micrognathia and hemifacial microsomia remains unclear; genetic elements are likely implicated, but data to date are controversial. The article “Transcriptome sequencing of facial adipose tissue reveals alterations in mRNAs of hemifacial microsomia” by Liu et al. reported that the increased expression of HOXB2 and HAND2 was associated with a facial deformity in children with HFM. Cell proliferation, migration, and invasion assays were performed with adipose-derived stem cells (ADSC) to confirm the phenotype of HOXB2. They found that the PI3K-Akt signaling pathway and human papillomavirus infection were activated in HFM. In conclusion, they discovered potential genes, pathways, and networks in HFM facial adipose tissue, which contributes to a better understanding of the pathogenesis of HFM.

Early and precise diagnosis for the patient with syndromic micrognathia is crucial to define. The most important message of “Prenatal diagnosis of micrognathia: a systematic review” by Cang et al. was that 15 biometric parameters related to the fetal mandible (IFA, FNMA, MNMA, MMFA, FMA, Mandibular Protrusion and Maxillary/Mandibular Protrusion, Mandibular Length, Jaw Index, MDW, Chin Index, Chin Length, Lower-facial Depth, Mandibular Curvature and Maxillary, FP line, Mandibular Gap Mandibular gap absence) could potentially provide simple and convenient diagnostic criteria or warning value for micrognathia. Based on these biometric parameters, clinicians could make a preliminary assessment of facial deformities and further clarify micrognathia by cytologic examination. “Measurement of the normal mandible in neonates in East China” by Jiang et al. reported data on mandibular measurements in a group of normal neonates in East China. The authors did not find any sex-dependent variation in mandibular size. Normal reference measurements of the mandibles in neonates were obtained. Three-dimensional reconstruction and measurements provided objective indicators for the diagnosis of micrognathia. In “Quantitative structural analysis of hemifacial microsomia mandibles in different age groups” the researchers underlined the importance of the quantitative structure of HFM mandibles in different age groups Zhang et al. The progression of asymmetry was assessed by changes in the affected/contralateral ratio with age using a multi-group comparison. Asymmetries were observed in the mandibular ramus/body regions. A significant contribution to progressive asymmetry in the body suggested a treatment focus in this region. These findings provide a basis for guiding the surgical management of HFM. Currently, minimally invasive robotic surgery hasbecome a focus of research in the treatment of syndromic micrognathia. In “Piezosurgery in hemifacial microsomia: a promising exemption from conventional peri-osteotomy suffering,” Wang et al. provided technical details relating to these techniques along with useful illustrations. The authors described the treatment of hemifacial microsomia using piezosurgery, which reduced intraoperative blood loss, operative time, and postoperative pain, providing an alternative to conventional osteotomy for the benefit of patients and families, and a more minimally invasive solution for HFM. Since 1992, the treatment of patients with HFM using mandibular distraction osteogenesis (MDO), has become an essential tool in the treatment of HFM. The continuous development of computer-assisted surgery (CAS) has laid the foundation for further improvements in surgical precision. CAS assists in preoperative design, intraoperative navigation, and postoperative evaluation, and has been applied to craniomaxillofacial surgery with great clinical value. Surgical robots have improved the inherent limitations of static navigation in craniofacial surgery. The manuscript “Accuracy and safety of robotic navigation-assisted distraction osteogenesis for hemifacial microsomia” by Zhang et al. verified the accuracy and safety of distraction osteogenesis for HFM using an Artificial Intelligence (AI) robotic navigation system.

Its clinical application needs to be further explored and validated.

In summary, this Research Topic provides original articles and reviews that add new information on the Pathogenesis, diagnosis, and management of Syndromic Micrognathia in the pediatric population. From pathogenesis to clinical management, the findings on this research topic were novel and useful. Notably, robotic navigation-assisted distraction osteogenesis ensures both the accuracy and safety of surgery. Additionally, it is our hope that these collected articles may spur additional research into these important topics.

